# Supplementary material for: Characterization of the Caudal Ventral Tubercle in the Sixth Cervical Vertebra in Modern Equus ferus caballus
Source: Animals (Basel). 2023 Jul 22;13(14):2384. doi: 10.3390/ani13142384 (PMC10376820; doi:10.3390/ani13142384)
Supplement: Supplementary file 1 [file animals-13-02384-s001.zip › animals-2456920-supplementary.pdf]

Supplementary Table S1. Details of the 76 osseous specimens that displayed an absent caudal ventral tubercle of the sixth cervical vertebra (only the osseous specimen was observed).

ID	Country	Age	Sex		ID	Country	Age	Sex
Tb1	AUS	5	M		Wb1	AUS	9	F
Tb2	AUS	6	M		Wb2	AUS	23	M
Tb3	AUS	16	F		Wb3	AUS	14	M
Tb4	AUS	18	M		Wb4	AUS	6	M
Tb5	AUS	5	F		Wb5	AUS	15	F
Tb6	AUS	12	M		Wb6	AUS	6	F
Tb7	AUS	12	F		Wb7	US	8	M
Tb8	AUS	10	M		Wb8	US	17	M
Tb9	AUS	4	M		Wb9	US	6	M
Tb10	AUS	12	F		Wb10	Netherlands	18	F
Tb11	AUS	7	F		Wb11	Netherlands	13	F
Tb12	AUS	22	F		Wb12	Netherlands	2	F
Tb13	AUS	10mths	M		Wb13	Belgium	SB	M
Tb14	AUS	12	M					
Tb15	AUS	8	F		ASH1	AUS	SB	F
Tb16	AUS	18	M		ASH2	AUS	3	M
Tb17	AUS	3	M		ASH3	AUS	5	M
Tb18	AUS	8	F		ASH4	AUS	22	F
Tb19	AUS	9	M		ASH5	AUS	10	M
Tb20	AUS	6	M		ASH6	AUS	8	F
Tb21	AUS	6	M					
Tb22	AUS	8	F		XB1	AUS	30	M
Tb23	AUS	9	M		XB2	AUS	12	F
Tb24	AUS	6	M		XB3	AUS	SB	M
Tb25	AUS	6	M		XB4	AUS	8	M
Tb26	US	9	F		XB5	NZ	15	M
Tb27	US	9	M					
Tb28	US	16	M		St1	AUS	13	M
Tb29	US	12	M		St2	AUS	12	M
Tb30	US	24	F		St3	NZ	22	M
Tb31	US	5	M		St4	Sweden	7	M
Tb32	US	20+	F					
Tb33	US	22	F		Ap1	US	23	F
Tb34	Japan	17	F		Ap2	US	22	F
Tb35	Japan	22	M		Ap3	US	35	M
Tb36	Japan	23	M					
Tb37	Japan	7	F		QH1	US	28	M
Tb38	UK	12	M		QH2	US	12	F
Tb39	Ireland	13	M					
Tb40	NZ	8	F		ISH1	UK	5	M
Fr1	US	10	M		RP1	AUS	8	M

Key: COUNTRIES: AUS – Australia; NZ – New Zealand; UK – United Kingdom; US – United States of America. BREEDS: Ap – Appaloosa; ASH – Australian Stock Horse; ISH – Irish Sport Horse; QH – Quarter Horse; RP – Riding Pony; St – Standardbred; Tb – Thoroughbred; Wb – Warmblood; XB – Cross Bred. AGE: SB – Still Born.

Supplementary Table 2. Individual grades of the 76 sixth cervical vertebrae selected for anomalous variations of the ventral process.

ID	Laterality	Unilateral L or R	aCVT Left	aCVT right	aCrVT left	aCrVT Right		ID	Laterality	Unilateral L or R	aCVT Left	aCVT right	aCrVT left	aCrVT Right
Tb1	Unilateral	L	4	0	2	0		Wb1	Unilateral	R	0	3	0	0
Tb2	Unilateral	L	4	0	2	0		Wb2	Bilateral		4	4	3	2
Tb3	Bilateral		4	4	3	3		Wb3	Unilateral	L	3	0	0	0
Tb4	Unilateral	L	2	0	0	0		Wb4	Bilateral		4	4	1	0
Tb5	Unilateral	L	4	0	2	0		Wb5	Unilateral	L	4	0	0	0
Tb6	Unilateral	R	0	1	0	0		Wb6	Unilateral	L	1	0	0	0
Tb7	Unilateral	R	0	4	0	0		Wb7	Unilateral	L	4	0	1	0
Tb8	Bilateral		4	4	1	1		Wb8	Bilateral		4	4	0	1
Tb9	Unilateral	L	4	0	3	0		Wb9	Bilateral		4	4	0	1
Tb10	Bilateral		3	4	0	0		Wb10	Unilateral	R	0	2	0	0
Tb11	Unilateral	L	4	0	2	0		Wb11	Unilateral	L	2	0	0	0
Tb12	Bilateral		4	4	0	0		Wb12	Unilateral	L	4	0	2	0
Tb13	Unilateral	R	0	4	0	2		Wb13	Bilateral		4	4	2	2
Tb14	Unilateral	L	2	0	0	0								
Tb15	Bilateral		4	4	2	1		ASH1	Bilateral		2	4	0	0
Tb16	Bilateral		4	4	0	0		ASH2	Bilateral		4	4	3	3
Tb17	Bilateral		4	4	0	0		ASH3	Unilateral	R	0	4	0	0
Tb18	Bilateral		4	4	0	0		ASH4	Unilateral	L	4	0	2	0
Tb19	Unilateral	L	4	0	2	0		ASH5	Unilateral	L	4	0	2	0
Tb20	Unilateral	L	4	0	2	0		ASH6	Unilateral	L	4	0	2	0
Tb21	Bilateral		4	4	2	2								
Tb22	Bilateral		4	4	0	0		XB1	Unilateral	L	4	0	2	0
Tb23	Unilateral	L	4	0	2	0		XB2	Bilateral		4	1	2	0
Tb24	Unilateral	L	4	0	2	0		XB3	Bilateral		4	4	0	0
Tb25	Bilateral		4	4	2	2		XB4	Unilateral	L	4	0	3	0
Tb26	Unilateral	L	4	0	2	0		XB5	Unilateral	R	0	3	0	0
Tb27	Unilateral	L	4	0	1	0								
Tb28	Bilateral		4	4	1	1		St1	Unilateral	L	1	0	0	0
Tb29	Unilateral	L	1	0	0	0		St2	Unilateral	L	2	0	0	0
Tb30	Unilateral	L	4	0	0	0		St3	Unilateral	L	2	0	0	0
Tb31	Bilateral		4	4	2	2		St4	Unilateral	R	0	2	0	0
Tb32	Unilateral	R	0	4	0	0								
Tb33	Unilateral	L	3	0	0	0		Ap1	Bilateral		4	4	2	1
Tb34	Unilateral	R	0	4	0	2		Ap2	Bilateral		4	4	1	0
Tb35	Unilateral	L	4	0	1	0		Ap3	Bilateral		4	4	0	0
Tb36	Unilateral	L	3	0	0	0								
Tb37	Unilateral	L	3	0	0	0		QH1	Unilateral	L	4	0	2	0

Tb38	Unilateral	R	0	2	0	0		QH2	Bilateral		4	4	1	2
Tb39	Bilateral		4	3	0	0								
Tb40	Unilateral	L	3	0	0	0		ISH1	Unilateral	R	0	2	0	0
Fr 1	Bilateral		4	4	2	3		RP1	Bilateral		4	3	1	0

Key: a – absent; ID – identification; L – left; R – right.
